# Current Trends in Artificial Intelligence for Recognizing Work Postures to Prevent Work-Related Musculoskeletal Disorders: Systematic Review and Meta-Analysis by Occupational Activity

**DOI:** 10.3390/bioengineering13030298

**Published:** 2026-03-03

**Authors:** Philippe Gorce, Julien Jacquier-Bret

**Affiliations:** 1 University of Toulon, CS 60584, Cedex 9, 83041 Toulon, France; gorce@univ-tln.fr; 2 International Institute of Biomechanics and Occupational Ergonomics, Avenue du Docteur Marcel Armanet, CS 10121, 83418 Hyères, France

**Keywords:** musculoskeletal disorders, human activity recognition, occupational activity, machine learning, deep learning, accuracy, precision, sensitivity, specificity, F1-score

## Abstract

The use of artificial intelligence (AI) to recognize postures is a promising approach for the prevention of work-related musculoskeletal disorders (WMSDs). The aim was to conduct a systematic review with meta-analysis to assess the performance of work posture recognition systems during occupational activity. The results were reported in accordance with the Preferred Reporting Items for Systematic Reviews and Meta-Analyses guidelines. The Google Scholar, IEEE Xplore, PubMed/MedLine, and ScienceDirect databases were screened without date restrictions. Two authors independently selected articles and extracted data. Studies were included if they presented a performance analysis of an AI deep learning (DL) or machine learning (ML) method that assessed the WMSD risk associated with working postures. Only peer-reviewed studies written in English including accuracy, precision, specificity, sensitivity, or F1-score values were included. The risk of bias was assessed using the Prediction Model Study Risk of Bias Assessment Tool. Of the 157 unique records, 58 studies were selected. The five performance parameters were investigated and averaged for seven occupational activities, eight posture categories, and the AI methods (ML vs. DL). Statistical analyses showed that DL methods produced better results. The reported systems detected sitting and standing postures with high accuracy. The solutions proposed in Manufacturing and Construction were the most numerous and the most effective on average. The major limitation lies in the wide variety of methods used. This analysis is a valuable source of information for designing new detection systems that are effective, ergonomic, easy to use, and acceptable so that humans remain at the center of the production process as defined by Industry 5.0.

## 1. Introduction

Musculoskeletal disorders (MSDs) are a major public health issue. A Global Burden of Disease analysis conducted in 2019 across 204 countries revealed that 1.71 billion people suffered from MSDs [1]. MSDs are characterized by inflammation and degeneration of bone, muscle, joint, and nerve tissue, leading to numerous pathologies or painful syndromes [2,3]. These disorders cause absenteeism, disability, and economic losses [4,5]. In Europe, MSDs accounted for 53% of all occupational illnesses and 50% of absences lasting more than three days. The total cost was estimated at around €240 billion, or up to 2% of GDP, which would represent 40–50% of the costs associated with all occupational health problems [6]. In the United States, more than 600,000 work-related musculoskeletal disorders (WMSDs) accounted for one-third of all lost workdays and generated costs of $54 billion, with direct annual costs related to treatment amounting to approximately $20 billion [5,7]. They are highly prevalent in many occupational activities such as healthcare [8], industry [9], construction [10], agriculture [11], office work [12], manufacturing [13], etc.

One of the major factors responsible for the occurrence of WMSDs is repeated and sustained awkward postures over long periods of time [14,15]. Several ergonomic tools can be used to determine the risk of WMSDs based on posture, the most commonly used being RULA (Rapid Upper Limb Assessment [16]) and REBA (Rapid Entire Limb Assessment [17]). These grids provide a risk score based on the position of different body segments of an individual in a working condition. However, these observational tools require the intervention of a specialized operator, focus on a specific posture or on posture extracts at specific intervals, and require analysis time [18,19]. As a result, they suffer from a lack of real-time feedback and continuous data collection, which are essential for proactive interventions covering the entire occupational activity.

The development of Industry 4.0 has enabled the implementation of automated equipment and digital solutions to increase productivity and workplace safety. The automation and digitization of industrial processes are characterized by the interconnection of systems such as cloud computing and the Internet of Things to automate decision-making and thus increase efficiency. At the same time, numerous policies aimed at reducing the incidence of WMSDs have been implemented to protect workers and improve their quality of life at work. Ergonomic interventions rely on the ability to detect and manage the risk factors to which individuals are exposed [20,21,22]. In this context, artificial intelligence (AI) is increasingly being used in the prevention of WMSDs due to its ability to process large quantities of human-related data. The development and improvement of multiple wearable sensors, vision-based techniques, and communication technologies have made it possible to continuously and automatically collect numerous physiological, muscular, or movement-related (kinematic, dynamic) parameters in real time [23]. Thanks to these databases and advanced algorithms, AI is now able to perform human activity recognition (HAR). HAR methodologies are designed to autonomously detect and classify individuals’ routine activities in defined contexts. Depending on the task and the technologies used, this can range from simple event detection to posture identification and classification. Sanchez-Comas et al. [24] proposed a review of work focused on HAR based on the 100 most cited studies. The areas of application cover generic activity recognition, posture recognition, and fall detection in the context of health and sports. Guerra et al. [25] provided a state-of-the-art overview of the different types of HAR sensors, highlighting the machine learning (ML) and deep learning (DL) methods. MLs rely on large databases to train different classifiers that are then used to recognize postures or activities. Support vector machines (SVMs) [26], artificial neural networks (ANNs) [27], k-nearest neighbors (KNN) [28], and decision trees (DTs) [29] are among the algorithms most commonly used by these methods. More recent DL methods identify, without significant human intervention, the characteristics necessary for detecting or classifying an activity from raw data [30]. The algorithms most commonly used by DL methods are convolutional neural networks (CNN) [31], long short-term memory (LSTM) [32], recurrent neural networks (RNN) [33], and encoders [34].

Numerous studies have used AI and its ML and DL methods in the context of WMSD prevention, and several systematic reviews have synthesized the results, highlighting different aspects. Çakit et al. [35] proposed an exhaustive bibliographic analysis (130 articles) on the use of ML for the assessment of WMSDs. Although the authors reported accuracy values, the objective was to identify gaps in research and provide guidance for future applications of ML in ergonomics. Shakerian et al. [36] published a review (46 studies) aimed at exploring how an artificial intelligence approach can be applied to manage the risk of WMSDs. Yang et al. [37] conducted a systematic review (30 studies) on advancing ergonomic posture risk assessment through the integration of computer vision and ML techniques. Finally, Donisi et al. [38] provided an overview of the most recent applications of AI (25 studies) and wearable sensors in physical ergonomics, as well as guidance on choosing the most appropriate wearable technology and AI strategy for ergonomic assessments and improvements in industrial and non-industrial environments. However, performance is often absent or rarely reported, even though it is crucial to the viability of an evaluation system. When constructing algorithms, authors evaluate performance using a confusion matrix that contains true and false positives, as well as true and false negatives, used to quantify parameters such as accuracy, precision, specificity, sensitivity, and F1-score [39,40]. Given the growing use of AI and the wide variety of solutions available, there are several effective algorithms for preventing WMSDs. However, the conditions under which these algorithms are used remain an important factor in their performance. This is because professional activities involve a wide range of different postures depending on the professional sector. These include sitting postures in office work, standing postures in industrial settings, and often awkward postures involving significant trunk flexion or rotation, or overhead activities in healthcare or construction. Because the proposed solutions are often tested in a well-defined context that does not necessarily include all of these postures, the evaluation of their performance remains linked to these conditions, and it can therefore be difficult to know which methods are most appropriate. Having an overview of the performance of existing AI solutions for the prevention of WMSDs according to the occupational activity and postures studied would be a real advantage for the protection of workers and their health at work.

Thus, the aim of this study was to propose a systematic review with meta-analysis to investigate the performance of artificial intelligence methods for recognizing work postures to prevent WMSDs. It was hypothesized that the diversity of the postures studied, the occupational activity, and the method and algorithms chosen had a direct impact on the overall system performance, i.e., on its ability to accurately detect WMSD risk based on a posture in the occupational environment. Performance was therefore assessed on the one hand using the parameters commonly presented in the literature, i.e., accuracy, sensitivity, specificity, precision, and F1-score, and on the other hand in terms of the number and diversity of postures considered when assessing the risk of WMSD. To meet this objective, this study proposes a classification of existing solutions based on their performance by occupational activity (office, construction, manufacturing, healthcare, etc.), by category of postures tested (sitting, standing, specific uncomfortable postures, etc.), and according to the AI method (ML vs. DL) and the classifiers chosen (SVM, KNN, CNN, LSTM, etc.). This analysis would provide answers to the following question: Is there a more effective approach to analyze a set of postures based on the occupational activity studied? Given the importance of preventing WMSDs, identifying the advantages of different solutions represents a major challenge for researchers, practitioners, and policymakers in the design and implementation of ergonomic solutions adapted to Industry 5.0.

## 2. Materials and Methods

The present systematic review and meta-analysis was conducted according to the PRISMA (Preferred Reporting Items for Systematic reviews and Meta-Analyses) guidelines [41,42] (see Supplementary Material for PRISMA abstract checklist [43]. The protocol was registered in PROSPERO under the number CRD420261284603.

### 2.1. Search Strategy

The objective of this research was to study the performance of posture identification algorithms with the aim of preventing the onset of musculoskeletal disorders in the workplace. The performance of the various systems is evaluated in the literature based on the following criteria: accuracy, sensitivity, and precision. To achieve this objective, a detailed list of keywords linked by the logical operators AND and OR was used in four open databases: PubMed/MedLine, Google Scholar, ScienceDirect, and IEEE Xplore. The search was conducted in December 2025 and all entries were considered without any date restrictions. Due to differences between search engines, the list of keywords had to be slightly adapted for each database. Table 1 details the search strategy for each database explored.

The selection of relevant articles was carried out in several stages. First, the results of the search conducted in each database were merged into a single Excel spreadsheet (Microsoft ^®^ Office Excel 2019, Redmond, WA, USA). Then a duplicate detection function was used. The evaluation of the articles was conducted in two steps by two independent reviewers (PG and JJB). An initial selection was made based on the title and abstract in relation to the inclusion/exclusion criteria. Any article that did not meet these criteria was excluded. The results obtained by each reviewer were compared in order to obtain the list of articles to be assessed based on their full text. All discrepancies were resolved by consensus. Finally, the full texts of remaining articles were evaluated and the list of articles obtained by each reviewer was compared to establish the final list of articles to be included in the analysis. All discrepancies were resolved by rereading the article and discussing it to reach a consensus, with the help of a third reviewer if necessary.

A second selection phase was conducted using the snowball method. During the evaluation of the full text of the articles, the reference list was analyzed. Any study that met the inclusion criteria was identified and then evaluated separately by two reviewers. Any studies that met the criteria were manually added to the list of included articles.

### 2.2. Selection Criteria

The inclusion of studies was based on several criteria. To be included, a study had to present a method for identifying posture in the prevention of work-related musculoskeletal disorders using artificial intelligence tools. The study also had to present one or more quantitative performance parameters for the proposed method, i.e., accuracy, precision, specificity, sensitivity, and F1-score. Only original studies that had been published and peer-reviewed were considered.

The following exclusion criteria were applied: (1) the study design was a conference, book or chapter, review, report, case report, or case study; (2) the study was not written in English; (3) the study had not been peer-reviewed; (4) the details of the method were insufficient; (5) the performance parameters were not available.

### 2.3. Risk of Bias Assessment

The Prediction Model Study Risk of Bias Assessment Tool (PROBAST [44]) was used to assess the risk of bias in the included studies. The assessment was conducted separately by the two reviewers (PG and JJB) and discrepancies were solved by consensus. PROBAST is structured as four domains with several items: participants (2 items), predictors (3 items), outcomes (6 items), and analysis (9 items). Each item was initially completed with the responses “yes,” “probably yes,” “probably no,” “no”, or “information missing.” For each item, the question was phrased so that “yes” indicates absence of bias. Based on the answers, each domain was rated as “low” (all responses were “yes” or “probably yes”), “high” (at least one element with a response of “no” or “probably no”), or “unclear” (at least one response of “information missing” and the others with a response of “yes” or “probably yes”) risk of bias. The overall judgment about the risk of bias of the prediction model was assessed according to the following rule: “low risk of bias” if all domains showed a low risk of bias, “unclear risk of bias” if an unclear risk of bias was found in at least one domain and a low risk in all others was set, and “high risk of bias” if at least one domain showed a high risk of bias. It should be noted that if the prediction model was developed without validation, it was assigned a high risk of bias. The results were presented in a traffic-light plot [45].

### 2.4. Data Extraction and Classification

For each article included, the following data were collected: name of the first author, year of publication, occupational activity, postures studied, method and algorithms used and their objective, measurement tools used and their position on the subject or in the environment, number of subjects tested, and all performance parameter values for the methods used. The methods refer to the two categories traditionally used in artificial intelligence literature: DL and ML [25]. All algorithms used by these two categories were reported (e.g., CNN, SVM, KNN, LSTM, DT, etc.) for each study. Finally, five performance parameters were selected: accuracy, precision, sensitivity, specificity, and F1-score (F1-score). These are obtained from the confusion matrix containing true positives (TP, correctly predicted positives), true negatives (TN, correctly predicted negatives), false positives (FP, incorrectly predicted positives), and false negatives (FN, incorrectly predicted negatives). The formulas used to determine these parameters are: accuracy = (TP + TN)/(TP + TN + FP + FN), sensitivity = TP/(TP + FN), specificity = TN/(FP + TN), precision = TP/(TP + FP), F1-score = 2 × (sensitivity × precision)/(sensitivity + precision).

### 2.5. Data Analysis

To achieve the objective of studying the performance of posture identification algorithms with the aim of preventing the appearance of WMSD, the studies were first classified by occupational activity. Performance analysis was carried out in four stages for each of the five parameters: according to occupational activities, postures adopted, artificial intelligence methods, and finally according to the algorithms used. In each analysis, the data available in the included studies were averaged.

### 2.6. Statistical Analysis

Statistical analyses were conducted to test the effect of occupational activity (wearable, not wearable, and hybrid solution) and methods (ML, DL) on performance parameters (accuracy, specificity, sensitivity, precision, and F1-score). Due to the non-normal distribution of data (Shapiro–Wilk test) and the non-homogeneity of variances (Levene’s test), a non-parametric Kruskal–Wallis test was used. Post hoc analyses were performed using Dunn’s test with Bonferroni-adjusted *p*-values. All analyses were performed using JASP software (JASP Team, v0.19.3, Amsterdam, The Netherlands), with a significance level set at 5%.

## 3. Results

### 3.1. Search Results

**Figure 1:**
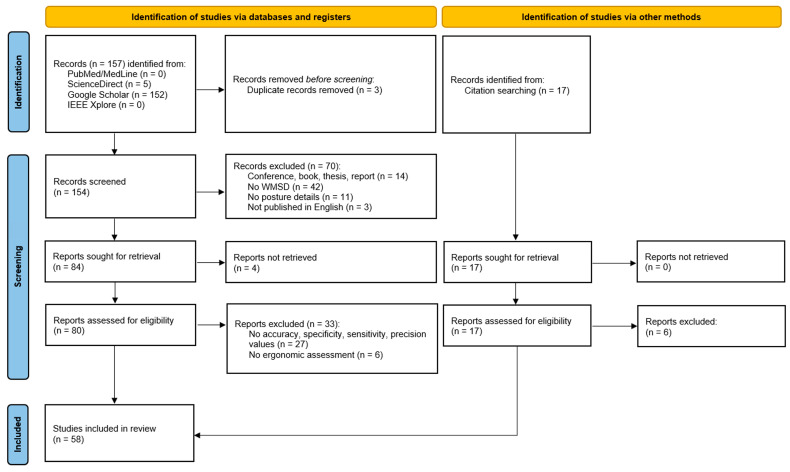
PRISMA flow diagram.

The search of the four databases identified 157 studies, with 3 of these being duplicates. Among the 154 unique articles, 107 were excluded because they did not meet the inclusion criteria: the format was not original peer-reviewed research, the algorithm evaluation was not conducted within the framework of WMSDs, the postures evaluated were not clearly detailed, or the algorithm performance parameters were missing. Following this selection, 47 articles were retained. The citation searching procedure identified 11 additional relevant articles. Thus, 58 articles were ultimately included in this analysis. Figure 1 illustrates the selection process.

### 3.2. Study Charactéristics

**Figure 2:**
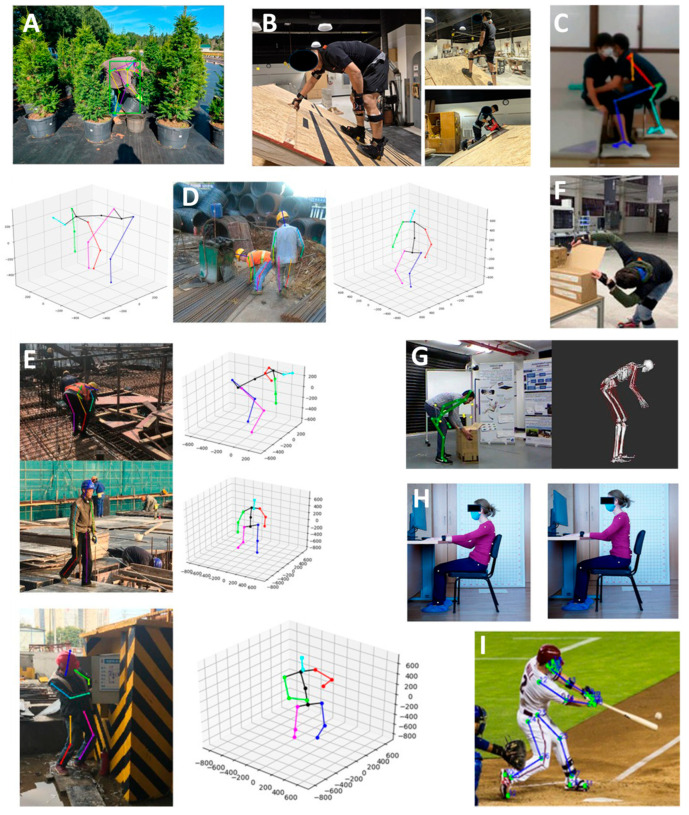
Examples of postures examined in different occupational activities. (**A**): Agriculture (standing with trunk flexed) [46]; (**B**,**D**,**E**): construction workers in general working posture (with 3 points of support, standing with feet staggered, kneeling) [47,55]; (**C**): healthcare professional during a transfer simulation [60]; (**F**,**G**): general working posture during manufacturing [67,69]; (**H**): office (sitting posture) [78]; (**I**): human activity during sport [84]. Some of the postures are accompanied by the modeling used by algorithms to study the risks of WMSDs.

Table 2 presents the studies included, providing information on occupational activity, postures studied, methods and algorithms used, measurement methods employed and their location, and the number of subjects involved in the evaluation process. Among the 58 studies, eight professional activities were identified: Agriculture [46] (one study), Construction [32,47,48,49,50,51,52,53,54,55,56,57] (12 studies), Healthcare [58,59,60,61,62,63,64,65] (eight studies), Industry [66] (one study), Manufacturing [29,67,68,69,70,71,72,73,74,75,76,77] (12 studies), Office [78,79,80,81,82,83] (six studies), Sport [84] (one study), and Transportation [85] (one study), in addition to 16 studies covering several professional activities. The latter were classified under the heading “All” [28,86,87,88,89,90,91,92,93,94,95,96,97,98,99,100]. The postures studied by the authors were classified into seven categories: general working posture [28,32,46,47,48,50,53,55,56,57,59,60,61,62,63,67,75,85,89,90,93] (19 studies), standing [58,61,64,66,86,91] (6 studies), sitting [29,73,78,79,80,81,82,83,87,88,92,94,95,98,100] (15 studies), manual handling [49,51,52,54,60,68,69,70,71,72,74,76,77,96] (14 studies), human activity [84,97] (2 studies), sitting and standing [99] (1 study), and standing and walking [65] (1 study). Figure 2 presents postures included in these different categories. In terms of methodology, machine learning (ML) was used in 30 studies [28,29,48,49,50,51,55,58,59,62,63,64,65,66,68,71,73,74,75,76,80,81,82,83,85,88,89,92,98,99] and deep learning (DL) was used in 21 studies [32,52,54,57,60,61,67,69,70,72,77,79,84,87,90,91,93,95,96,97,100]. Seven studies proposed solutions using these two approaches [46,47,53,56,78,86,94]. A large number of algorithms were used in the 58 studies. Convolutional neural networks (CNNs) and long short-term memory (LSTM) were the most frequently used in DL, while support vector machines (SVMs) and k-nearest neighbors (KNN) were the most frequently used in ML. With regard to sensors, both portable and non-portable solutions were identified. For portable solutions, surface electromyography (EMG), inertial measurement units (IMUs), foot pressure sensors, and physiological sensors (temperature, respiration) were mainly used. For non-portable solutions deployed in the environment, the authors used different types of cameras (RBG, depth, optoelectronic), or various pressure or force sensors. Several studies also used questionnaires, often standardized, to collect other information about the subjects necessary for the implementation of the proposed solution. The majority of studies tested their solution on a set of experimental data.

Table 3 presents the performance parameters computed in each study, classified by occupational activity. The presence of the symbol X indicates that the authors evaluated their solution using the parameter considered. Accuracy was the most frequently used by the authors (50 studies) and specificity was the least commonly reported (12 studies). Twenty-two studies used only one of the five parameters to evaluate their solution, while only five studies computed all of them. The remaining 16 studies mainly used four parameters. Given that the authors often proposed several alternatives and several evaluations, the total number of entries available for the present performance analysis was 778. Thus, the quantities of data analyzed were: 552 accuracy values from 50 studies, 157 specificity values from 12 studies, 344 sensitivity values from 31 studies, 277 precision values from 25 studies, and 374 F1-scores from 31 studies.

### 3.3. Risk of Bias

**Figure 3:**
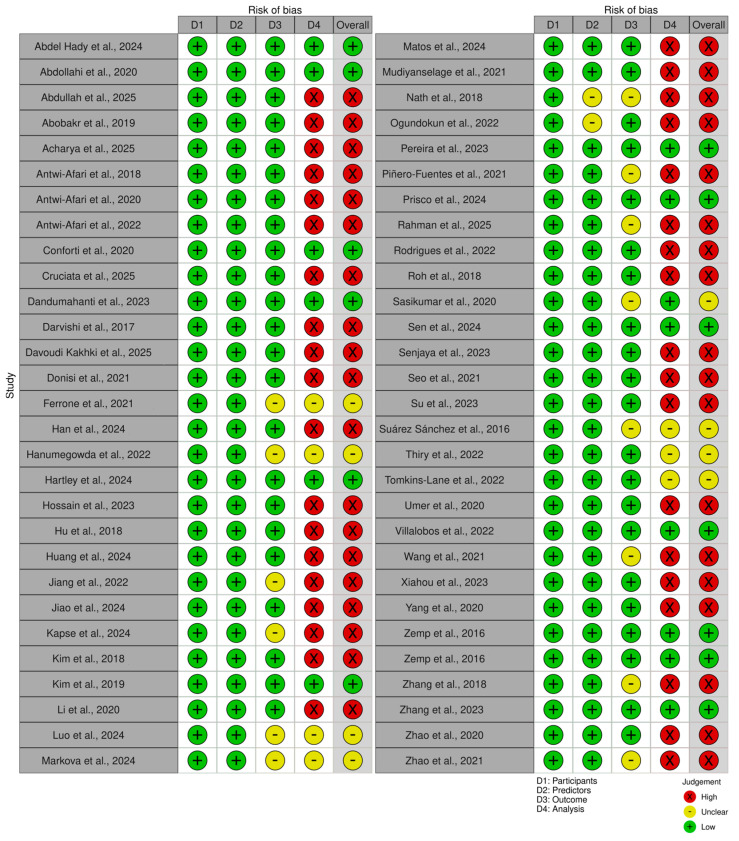
Traffic-light plot of the risk of bias. References [28,29,32,46,47,48,49,50,51,52,53,54,55,56,57,58,59,60,61,62,63,64,65,66,67,68,69,70,71,72,73,74,75,76,77,78,79,80,81,82,83,84,85,86,87,88,89,90,91,92,93,94,95,96,97,98,99,100].

Figure 3 presents the results of the risk of bias analysis for the included studies. Thirteen studies had a low risk of bias, while 37 were scored with a high risk of bias. An unclear risk of bias was assigned to the remaining eight studies. The main reasons that led to a high risk of bias were the lack of validation of the predictive model or too small number of subjects.

### 3.4. Results of Posture Recognition Performance per Occupational Activity

**Figure 4:**
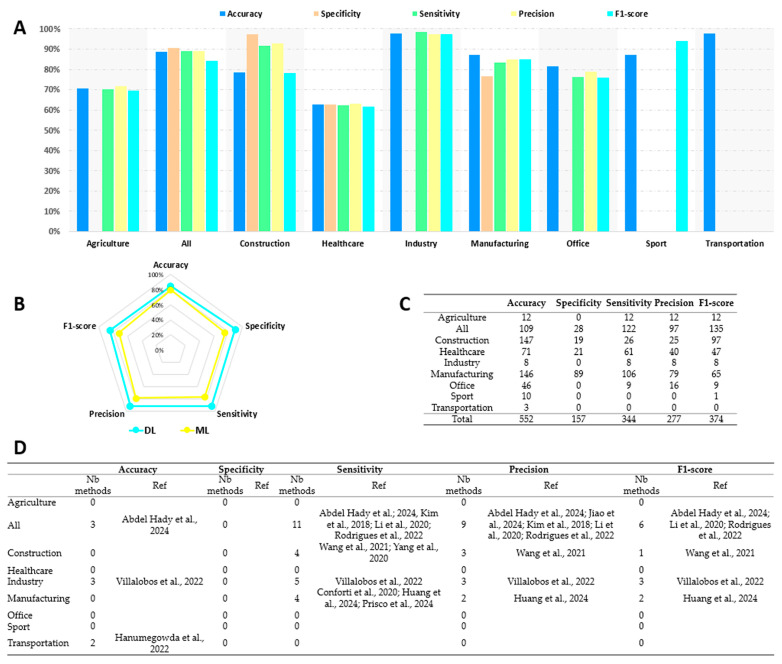
Performance analysis based on occupational activity. (**A**): Histogram showing the values of the five performance parameters by occupational activity. (**B**): Radar chart comparing ML and DL for the five performance parameters. (**C**): Table showing the number of values available for each performance parameter by occupational activity. (**D**): Table displaying the distribution of studies that achieved 100% performance by parameter and occupational activity. DL= Deep Learning; ML = Machine Learning; Nb = number; Ref = references. References: [52,54,66,68,72,76,85,86,93,94,96,99].

Figure 4 presents the performance parameters analysis by occupational activity. Parts A and B detail the values of the performance criteria by occupational activity and by method, Table C indicates the number of available values analyzed, and Table D presents the number of methods that achieved 100% performance.

The statistical analysis revealed differences between professional activities for all performance parameters. First, Transportation (97.7%) and Industry (97.7%) were the professional activities with the highest accuracy (F = 102.6, *p* < 0.05, ε^2^ = 0.186), followed by All (88.8%) and Manufacturing (87.2%). These values were statistically different from those obtained for Office (81.5%), Construction (78.7%), Agriculture (70.7%), and Healthcare (62.8%, the lowest accuracy observed).

Specificity was the parameter with the smallest number of available data. As a result, the analysis could only include four occupational activities (F = 51.4, *p* < 0.05, ε^2^ = 0.330). Among these, Construction (97.4%, *p* < 0.05) performed best, while Healthcare (62.7%) performed worst. All (90.4%) and Manufacturing (76.8%) were the other two activities, but were not statistically different.

Industry (98.4%), Construction (91.6%), and All (89.1%) achieved the highest sensitivity compared to Manufacturing (83.5%), Office (76.3%), Agriculture (70.3%), and Healthcare (62.2%, F = 74.1, *p* < 0.05, ε^2^ = 0.216).

Industry (97.7%) and Construction (97.7%) were the professional activities with the highest accuracy, followed by All (88.8%) and Manufacturing (87.2%). For these four professional activities, the values were statistically higher than for Agriculture (71.8%) and Healthcare (62.9%, *p* < 0.05). Industry (97.7%) and Construction (97.7%) also provided greater accuracy than Office (78.7%, F = 54.6, *p* < 0.05, ε^2^ = 0.198).

The F1-scores for Industry (97.3%) and Manufacturing (85.0%) were statistically different from the F1-scores found for Office (75.8%), Agriculture (69.3%), and Healthcare (61.4%). All (84.0%) and Construction (78.2%) obtained an intermediate F1-score (F = 48.3, *p* < 0.05, ε^2^ = 0.130).

No sensitivity, precision, or F1-score values were available for Sport and Transportation.

Regarding the methods used, the performance of DL methods was statistically higher than the performance of ML methods respectively for accuracy (84.9% vs. 79.9%, *p* < 0.05), specificity (89.8% vs. 75.5%, *p* < 0.05), sensitivity (91.2% vs. 76.2%, *p* < 0.05), precision (91.4% vs. 78.5%, *p* < 0.05), and F1-score (84.9% vs. 72.4%, *p* < 0.05).

### 3.5. Posture Recognition Performance per Occupational Activity and AI Method

**Figure 5:**
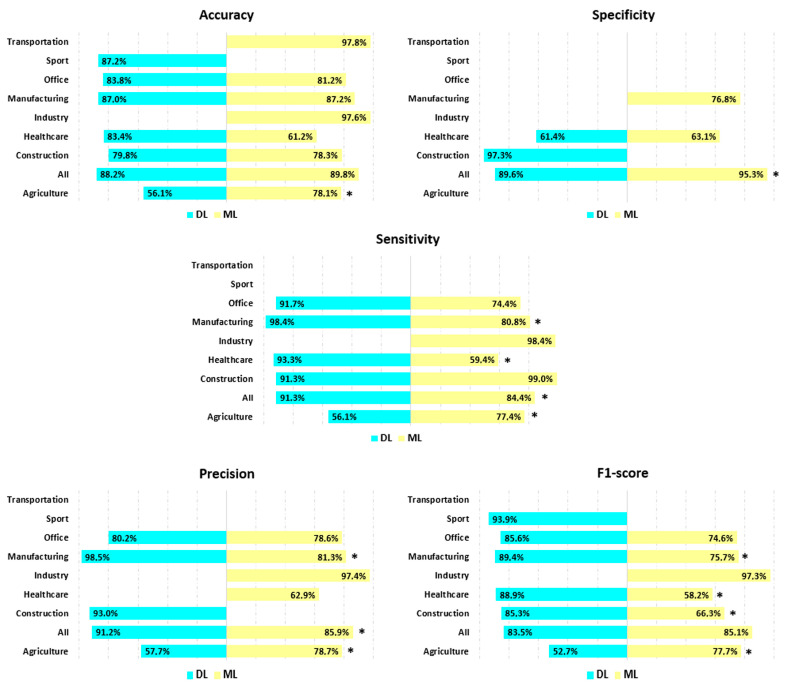
Performance analysis by professional activities and AI methods. * represents a significant difference in performance between the two AI methods (*p* < 0.05).

Figure 5 shows the comparison of performance obtained by deep learning and machine learning methods for each of the parameters by professional activity. For Agriculture, the accuracy of ML methods was higher than for DL methods, while sensitivity, precision, and F1-score were higher for DL methods. For All, specificity was also higher for ML methods than for DL methods, while sensitivity and precision were higher for DL methods. For sensitivity (Healthcare and Manufacturing), precision (Manufacturing), and F1-score (Construction, Healthcare, Manufacturing), DL methods consistently outperformed ML methods.

### 3.6. Posture Recognition Performance per Posture Studied During Occupational Activities

**Figure 6:**
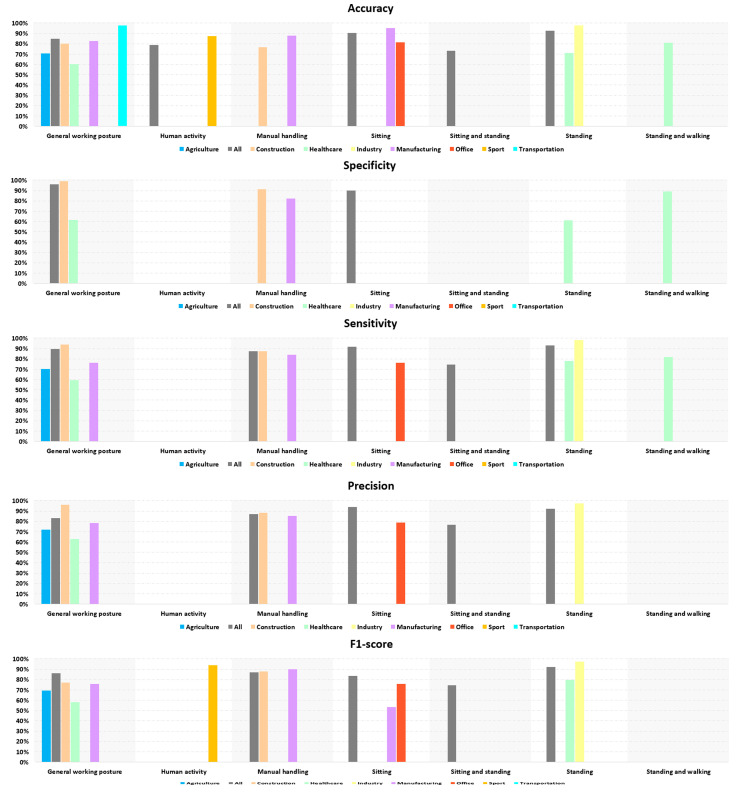
Performance analysis based on occupational activities and postures studied.

Figure 6 shows the performance of detection methods by posture category observed in different professional activities. For all postures combined, performance varies between 60% and 99% for accuracy, specificity, sensitivity, and precision, and between 50% and 98% for F1-score. The performance of the General working posture was the most investigated category in the included studies. The proposed solutions achieved very good performance in Construction for specificity, sensitivity, and precision, with values greater than or equal to 95%. Equivalent performance was observed for accuracy in Transportation and specificity in the All category. Specificity in Construction for the Manual handling posture category also performed well (91.3%).

Standing posture detection solutions performed very well for the All activity, i.e., accuracy, sensitivity, precision, and F1-score between 90% and 95%, and even better in Industry with values above 95% for these four parameters.

The Sitting posture detection methods also provided very good results for All, with accuracy, specificity, sensitivity, and F1-score between 90% and 95%, as well as in Manufacturing with an accuracy of 95%.

### 3.7. ML and DL Algorithm Performance per Occupational Activity

Figure 7, Figure 8, Figure 9, Figure 10, Figure 11, Figure 12, Figure 13 and Figure 14 display the performance of the algorithms that were most frequently used by occupational activity.

In the field of Agriculture, the four ML algorithms, i.e., SVM, RF, DT, and ANN, achieved values of approximately 80% for accuracy, sensitivity, precision, and F1-score. For DL, Inception, MobileNet, and VGG16 performed at around 60% for these same four parameters. Only ResNet demonstrated lower performance (approximately 40%).

In the field of Construction, ML algorithms were tested only for accuracy, sensitivity, and F1-score. For accuracy, EC and BP-ANN had the best scores (99%), while ANN, Bagged Tree, and Discriminate were the least effective (<60%). For F1-score, SVM and Hybrid Transformer were the two best algorithms (around 80%). In DL, CNN and GRU performed very well with values above 95% for specificity, sensitivity, precision, and F1-score. For accuracy, the highest values were obtained with the RNN and Bi-LSTM algorithms (85%).

Posture recognition in the Healthcare field has mainly been achieved using ML methods. The highest accuracy was obtained with the NB and RF algorithms (80%). For the other algorithms and parameters, performance was mostly between 60 and 70%. Only the CNN algorithm was used in DL methods, with performance varying depending on the performance parameter.

In Industry, only four ML algorithms were used, i.e., DT, ET, RF, and SVM, with performance above 95% for all performance parameters (except specificity, which was not evaluated).

For Manufacturing, SVM was the most widely used ML method algorithm, with an overall performance of 80%. GB, DT, and RF performed very well, with values of at least 90% for all five parameters. NB and PNN were the least effective overall, with values below or equal to 80%. For DL methods, Spectre-Vit and MLP achieved the best performance (>95%) for the four parameters evaluated (no values for specificity), as did CNN (except for accuracy). The LSTM algorithm performed the worst overall, but still achieved values of 90% for the four parameters.

During Office activity, the performance (except for specificity, which was not evaluated) of all the algorithms used was around 80%, with the highest values observed for RF. SVM was the least effective algorithm, with values below 40% for sensitivity, precision, and F1-score. Similar performance was observed for DLNN and CNN, the only two algorithms used in DL.

In the study focusing on Sports, only a DL approach was used, and the proposed algorithms achieved accuracy values between 85% and 90%. Visk-Gat presented the highest accuracy value (93.9%) and the only F1-score value (93.9%).

In Transportation, only accuracy values above 95% were reported for three proposed ML methods, i.e., DT, BN, and RF.

In the context of general working postures not assigned to a specific occupational activity (All), the KNN and RF algorithms were the most widely used in ML, and CNN and LSTM in DL. RF and DT performed the worst in ML (around 80% for sensitivity, precision, and F1-score), while KNN, SVM, NC, and GMM achieved performance above 90%. In DL, the CNN, LSTM, encoder, and GMU algorithms scored close to or above 90%. The accuracy of MLP, the precision of DNN, and the F1-score of DNN and HOG were less effective, with values below 80%.

## 4. Discussion

The objective of this systematic review was to provide an overview of the recognition of work postures using artificial intelligence in order to prevent the onset of WMSDs. Knowledge of their performance is essential to select the most appropriate method for a professional activity, considering its various constraints. The analysis was carried out considering the working postures adopted and the different methods and algorithms based on ML and DL according to each occupational activity encountered in the 58 studies included.

### 4.1. Posture Recognition Performance per Occupational Activity

The analysis identified eight occupational activities for which the number of available studies varied greatly. The most prolific fields were Construction, Manufacturing, Healthcare, and Office. Construction was the best-performing field, with three of the five parameters (specificity, sensitivity, and precision) exceeding 90% and the other two being close to 80%. For Manufacturing, accuracy, sensitivity, precision, and F1-score all scored above 80% (with specificity at 75%). For Office, specificity was not studied. For the other parameters, only accuracy reached 80%, while the other three were around 75%. Healthcare was the domain with the lowest performance, with values of approximately 60%. Despite the diversity of activities, All performed well with an F1-score of 85% and the other four parameters close to 90%. However, these interesting results should be viewed with caution, as solutions in each of these areas perform very well. Indeed, Manufacturing, Construction, and All presented solutions with 100% performance (Figure 4, Table D) [52,54,68,72,76,86,93,94,96,99]. For Healthcare, two studies reported performance levels of around 95% for four parameters. For Office, Markova et al. [78] identified a system with a performance level of 85% for four parameters. All these results lead to the identification of the choices and assumptions made by researchers in developing their solution, which could have a significant impact on its performance. First, the number of body segments involved is a major assumption. Indeed, the risk of WMSDs was studied from the perspective of a single joint or the entire body. Thus, Thiry et al. [64] and Abdollahi et al. [58] studied the prevalence of low back pain through trunk flexion/extension, while Wang et al. [52] and Han et al. [60] considered all joint angles (3D model with 14 or 25 key points respectively). The method used to assess WMSDs is also important. Some authors assessed this risk by classifying postures as safe or unsafe [76]. Other authors assessed the overall posture and then used a standard ergonomic tool such as RULA or REBA [72,99]. The nature of the sensors used is also a source of performance variation [101]. Across all of the studies included, a wide variety of sensors were observed. The wearable sensors identified were mainly IMUs, foot pressure sensors, and physiological sensors (ECG, EMG, EEG, etc.), while non-wearable solutions mainly used different types of cameras (RGB, depth, etc.). For these two categories, the results showed performance variability ranging from 20% (wearable in Healthcare [63]; non-wearable in Agriculture [46]) to over 90% (wearable in Industry [66]; non-wearable in Construction [52]). The same results were evidenced for hybrid studies combining these two categories (60% in Healthcare [58] to over 98% in Manufacturing [69]). The type, number, positioning, and performance of sensors do not offer the same potential for posture assessment. With a small number of sensors or a single-plane assessment, posture is only modeled in 2D [64], whereas more advanced combinations allow for 3D estimations [72]. The information available for assessing WMSDs is therefore different, which may be the cause of the performance variations observed. All of this suggests that work should be done to further evaluate the performance of the proposed methods so that it is more consistent and therefore objective.

### 4.2. ML vs. DL Performance for Posture Recognition in Occupational Activity

In the context of WMSD prevention addressed in this study, the results showed that DL algorithms performed significantly better than ML algorithms for the five parameters tested. These results are consistent with other studies carried out in the Healthcare field [102,103]. Vu Thi Thu et al. [102] reported that CNN and LSTM (DL method) were more effective for real-time gait recognition in robotic prostheses, mainly in terms of accuracy. Korotcov et al. [103] found that DL networks outperformed ML algorithms using SVMs in pharmaceutical research using seven performance parameters. However, other studies have highlighted the superiority of ML algorithms. For example, in the field of human activity recognition, Baldominos et al. [104] showed that ensembles of decision trees (i.e., extremely randomized trees and random forests) achieved the best accuracy. In the field of physics, Hafsa et al. [105] showed that extreme gradient boosting was identified as the optimal model for predicting two-phase flow regimes in inclined or horizontal pipes using five performance parameters.

The analysis by profession shows the best performance for DL algorithms for the various parameters. For Construction (12 studies), Healthcare (8 studies), and Manufacturing (12 studies), the studies included tested their solution on a large number of different postures (sitting, standing, walking, manual handling with different weights or normal work vs. overhead, or general working posture, e.g., lying down, kneeling, etc.) including several pieces of information (several segments, several muscles, multiple types of data) to assess the risk of WMSD. It is well known that DLs are more suitable for complex applications requiring a large amount of data. This complexity in the evaluation of WMSDs is often necessary, particularly for the use of ergonomic assessment tools such as RULA or REBA, which require a great deal of joint and environmental information [16,17]. This could explain the better performance observed for DL methods in these three areas [52,60,72].

For Office (six studies), no difference was found between ML and DL. Finally, accuracy in Agriculture and specificity for All were higher for ML algorithms. However, the number of studies for which data were available was very low (only 1 to 2 studies), which does not allow these observations to be generalized, as is the case for Sport, Transportation, and Industry, where the quantity of data was insufficient.

### 4.3. Performance per Posture Studied During Occupational Activities

The various occupational activities were represented by eight categories of postures. General working posture was observed in the majority of occupational activities. Manual handling also encompasses several postures and was mainly observed in Manufacturing and Construction. Other more specific postures were only observed in some activities, such as standing and walking in Healthcare or sitting in the Office. This shows that there appear to be specific postural characteristics depending on the field. However, the fields of Sport, Transportation, Agriculture, and Industry are too underrepresented to be able to generalize this emerging trend.

In regard to performance parameters, accuracy was the most commonly used parameter across all occupational activities. Conversely, specificity was rarely reported. In terms of values, it appears that posture recognition algorithms perform fairly well for well-defined postures such as sitting and standing, with values above 90% (accuracy, sensitivity, and precision) for Manufacturing, Industry, and All. Only Healthcare seems to be an area where the methods perform less well with the standing posture. When several postures are considered in studies, performance results (all performance parameters combined) tend to decrease (between 70 and 80% on average). To our knowledge, no study has proposed a classification of performance parameters by occupational activity. It is therefore difficult to position these results in relation to the literature. However, it is a promising avenue for classifying methods according to their performance and the specificities of occupational activities.

### 4.4. ML and DL Algorithms Performance per Occupational Activities

Analysis of the various solutions proposed in the studies included revealed that a large number of algorithms were used for both ML and DL methods. The most commonly used algorithms for these two categories are CNN and LSTM for DL, and DT, RF, KNN, and SVM for ML. However, the distribution of algorithms and their performance varied greatly depending on the occupational activity. The three occupational activities with the highest number of studies were Construction, Healthcare, and Manufacturing.

In the field of Construction, many different algorithms have been used for ML and DL. The performance of ML algorithms has been less well evaluated. Only accuracy and F1-score have been presented. DL algorithms have been evaluated with all parameters. CNN and LSTM have shown very interesting performance values above 90% (but with lower accuracy). Similar results were observed with these algorithms in different fields such as fall detection [106], skin cancer detection [107], and classification of diabetic retinopathy [108]. For this occupational activity, analysis using DL methods has proven to be predominant and shows superior performance results for the recognition of manual handling postures [52] or for different postures (kneeling, standing with arms raised above the head, etc.) [32].

In the Healthcare field, the trend is reversed and ML algorithms have been predominant. None of the ML algorithms proposed performed better than 90%. For the DL part, only CNNs were used and achieved a performance of around 90% for sensitivity and F1-score. Further investigation is needed in this field due to the small number of studies and the overall performance, which still has potential for improvement (60–80%).

In the Manufacturing field, a large number of algorithms have been proposed using ML and DL methods. For the DL part, the majority of algorithms achieved performance levels above 90% for accuracy, sensitivity, precision, and F1-score (no spec evaluation was proposed). For the ML part, all parameters were evaluated with variable performance ranging from 60% to over 95%. GB, DT, and RF were the most effective for identifying postures, with performance above 90%. Based on this summary, it appears that algorithms derived from both methods allow for a good assessment of WMSD risks.

For Office, ML algorithms were much more numerous than DL algorithms. Performance in this area was around 80% for the accuracy, sensitivity, precision, and F1-score parameters (no specificity evaluation was proposed). It should be noted that the SVM algorithm, although very effective in other areas, was the one that performed the worst. In this area, the most studied postures are sitting postures, where the aim is to distinguish between different variants [78,82], i.e., leaning to the side, sitting against the backrest, leaning forward, etc. The lower performance of the proposed solutions can be explained by a dichotomy of the sitting posture into multiple sub-postures differentiated by a small angular variation between them.

The postures were classified using many algorithms for ML and DL, the most common being CNN and LSTM for DL, and DT, RF, KNN, and SVM for ML. The overall accuracy of all these algorithms was around 90%, while the values of the other parameters were more variable (ranging from 60% to 95%). The best algorithms were CNN, LSTM, KNN, and SVM, which offered performance of around 90% for all parameters and therefore appear to be good tools for detecting different working postures.

### 4.5. Limitations

The first limitation of this study concerns the quality of the results reported in the included studies. Although several algorithms demonstrated good performance, the lack of validation or the insufficient number of subjects limits the generalizability of the results. Furthermore, differences in the definitions of the WMSD risk level were observed, which exacerbates this issue. Indeed, some studies evaluated their solution based on postures defined as good or bad, while other studies used different ergonomic assessment tools to evaluate the risk of WMSDs (RULA for the upper body vs. REBA for the whole body). The use of these ergonomic tools requires the intervention of an expert to establish the comparative data and this part is not always defined in studies.

The second limitation concerns the conditions for evaluating the performance of posture detection systems. In most studies, solutions were evaluated using data collected as part of specifically developed experimental protocols, in which the subjects or conditions could vary greatly between studies. In addition, the nature (sitting or standing), number, and complexity of the postures (number of segments or features considered by the model) were also very different. As a result, the datasets were very heterogeneous. This has a significant impact on the evaluation of solution performance, regardless of the sensors, methods, and algorithms used. This heterogeneity also affects the training part of machine learning (ML) and deep learning (DL) methods, which in turns affects the performance parameters of the method being studied. It therefore seems important and necessary to standardize the evaluation conditions in order to be able to accurately compare the various studies. Finally, even though the datasets used have improved, they still only offer stereotypical posture solutions without taking into account the anthropometry, gender, age, or health profile of the operators, or variations in the environment.

The third limitation concerns performance evaluation. It was conducted by occupational activity, integrating all the proposed solutions. However, it has been shown that the type of system—wearable, non-wearable, or hybrid—can directly impact the detection performance of AI algorithms depending on the situation. One solution could therefore be to extend the analysis by breaking it down into subgroups of sensor families or by specifically comparing algorithms of the same type (SVM, KNN, RF, DT, CNN, LSTM, etc.).

Another limitation concerns the number of studies available overall and by occupational activity (the most prolific activities were Construction (12 studies), Manufacturing (12 studies), and Healthcare (8 studies)). This limited number had an effect on the number of performance parameters available and their distribution by method (ML vs. DL) and by algorithm.

The final limitations are methodological and relate to the inclusion/exclusion criteria for articles. On the one hand, the research focused on posture detection systems during occupational activities using specific keywords, without necessarily using all their synonyms. On the other hand, only original, peer-reviewed research written in English was included. These choices may have led to the omission of some studies that could have enriched the systematic review.

### 4.6. General Outcomes and Future Research Directions

As illustrated by this systematic review and meta-analysis, posture detection systems offer effective solutions, with an overall effectiveness (all parameters combined) of approximately 90% in several occupational activities. Some solutions even achieve 100% for several parameters. Despite this, these detection systems remain imperfect and require further investigation, particularly in terms of the definition, representation, and classification of postures. The methodological aspect must be strengthened (validation procedure, minimum number of subjects, etc.) in order to guarantee the quality of the model’s results and its widespread use in one or more occupational activities. It is particularly important to link a model’s performance to its ability to analyze a wide variety of postures in different contexts. In the field of WMSD prevention, it seems relevant to distinguish between models based on the recognition of postures previously classified as good or bad and solutions that incorporate recognized ergonomic tool standards such as RULA or REBA. Future advances could focus on customizing detection systems based on operator-specific data (anthropometry, gender, age, health profile, etc.). Systems should therefore incorporate more sophisticated predictive algorithms in order to anticipate the onset or maintained use of awkward postures and take preventive measures, for example by warning the operator.

To achieve this, when using wearable sensors, it is important to ensure that they do not interfere with workers’ movements or productivity. Acceptability is also a key issue, as future solutions will need to guarantee lasting comfort, good ergonomics, and usability. This can only be achieved by designing intuitive, personalized interfaces and lightweight, miniaturized solutions specific to the occupational activity [109]. The development of smartwatches, smartphone apps, and, more recently, smart clothing, are the first solutions to be proposed [75,92]. With regard to non-wearable sensors and hybrid solutions, privacy must be taken into account by addressing ethical and social acceptability issues and constraints. The Internet of Things (IoT) represents a promising issue for posture detection in the context of WMSD prevention [110]. All these areas of research and development will lead to Industry 5.0, which aims to integrate artificial intelligence, automation, and robotics into human-centered production processes [111].

Establishing reliability and security standards for these new systems is an important area of research. This would lead, for example, to the creation of a list of universal performance parameters within a standardized common framework, enabling solutions to be compared objectively. The development of secure data transmission and storage methods is also essential to protect user privacy.

All these advances could result in the development of adaptive and high-performance systems able to consider users’ health profiles and professional activities in order to meet the objectives of Industry 5.0.

## 5. Conclusions

This literature review and meta-analysis provided an overview of posture recognition systems for the prevention of WMSDs. Its originality resides in the performance comparison of the two main AI methods, i.e., ML vs. DL, and their algorithms for six professional activities and seven families of working postures. The results showed that manufacturing and construction were the activities with the most effective methods. Across all occupational activities, DL methods showed the highest performance parameters, as did the WMSD risk assessment methods for sitting and standing postures. This analysis is a valuable source of information for improving or designing new detection systems that are more effective and better suited to both the specificities of the activity and the workers. By integrating these performance aspects with ergonomics, acceptability, privacy, and productivity, future solutions could help ensure that humans remain at the center of the production process as defined by Industry 5.0.

## Figures and Tables

**Figure 7:**
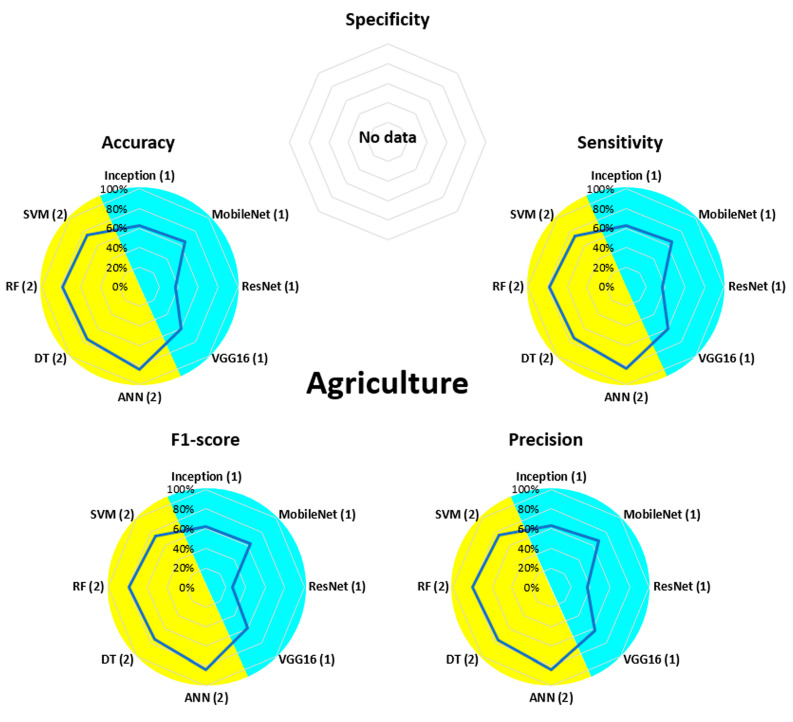
Performance analysis of different algorithms used in ML (yellow) and DL (blue) methods in Agriculture.

**Figure 8:**
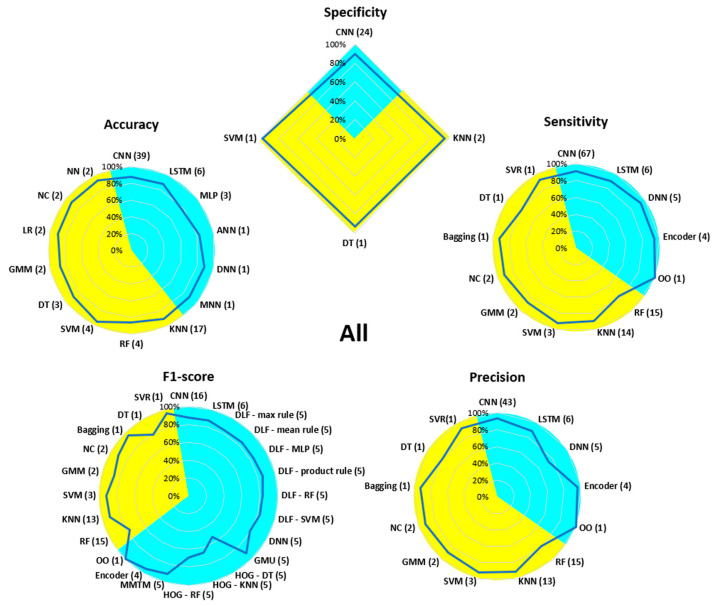
Performance analysis of different algorithms used in ML (yellow) and DL (blue) methods in All.

**Figure 9:**
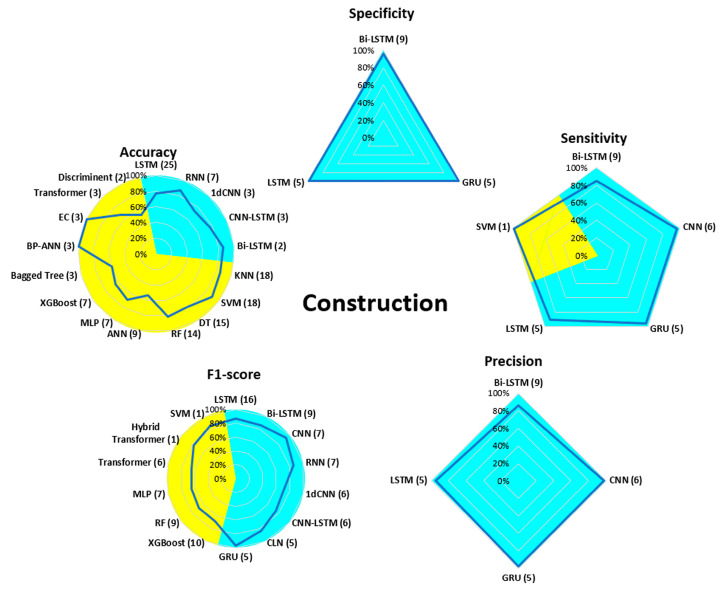
Performance analysis of different algorithms used in ML (yellow) and DL (blue) methods in Construction.

**Figure 10:**
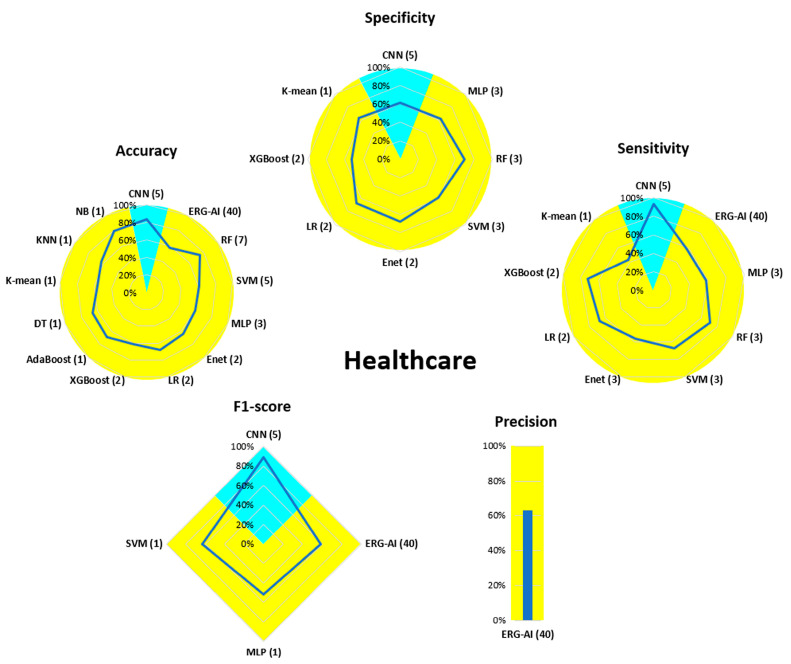
Performance analysis of different algorithms used in ML (yellow) and DL (blue) methods in Healthcare.

**Figure 11:**
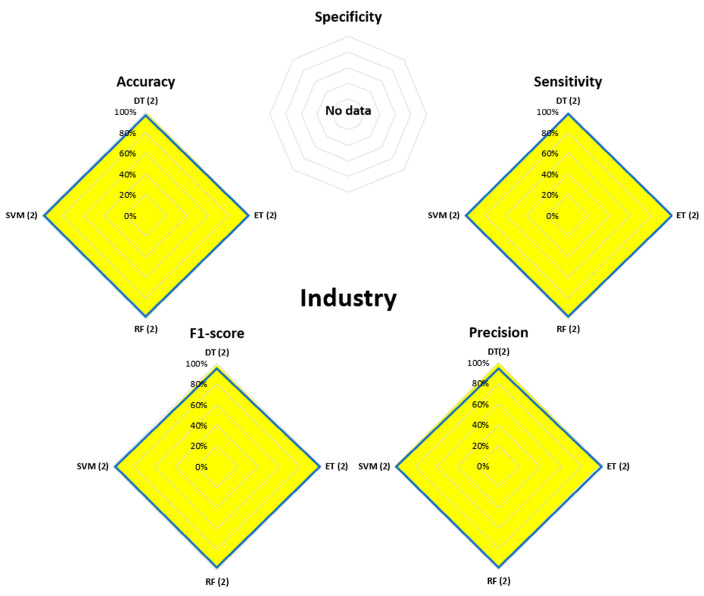
Performance analysis of different algorithms used in ML (yellow) methods in Industry. No DL methods were used.

**Figure 12:**
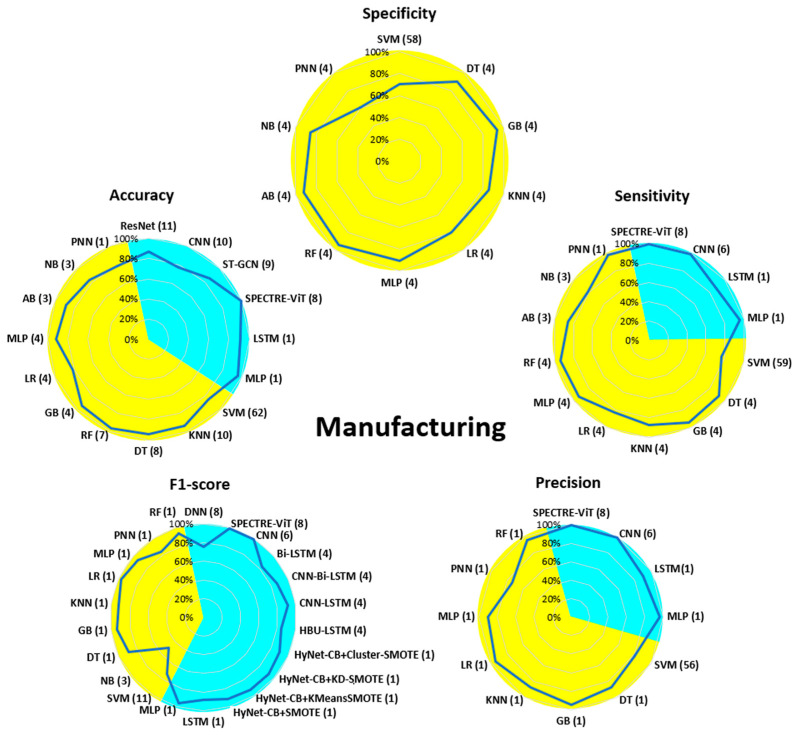
Performance analysis of different algorithms used in ML (yellow) and DL (blue) methods in Manufacturing.

**Figure 13:**
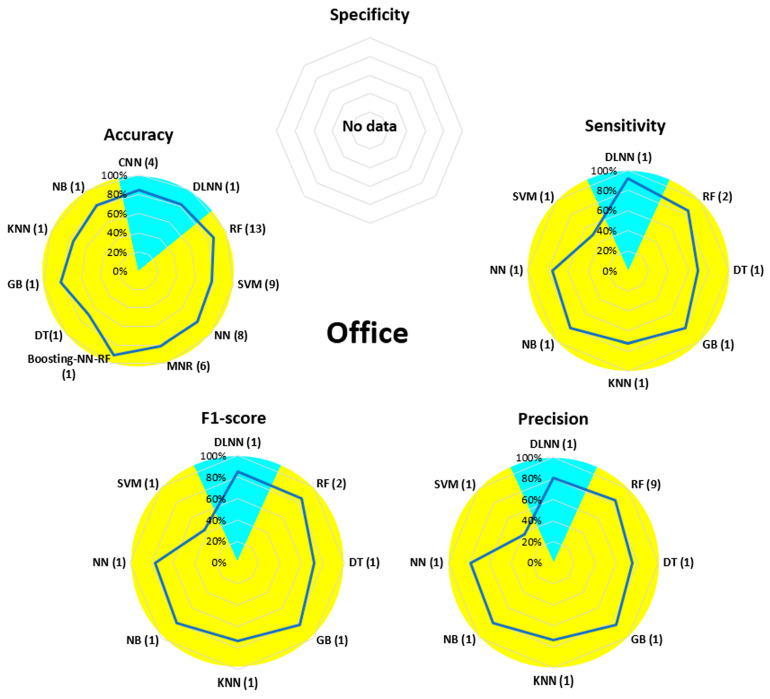
Performance analysis of different algorithms used in ML (yellow) and DL (blue) methods in Office.

**Figure 14:**
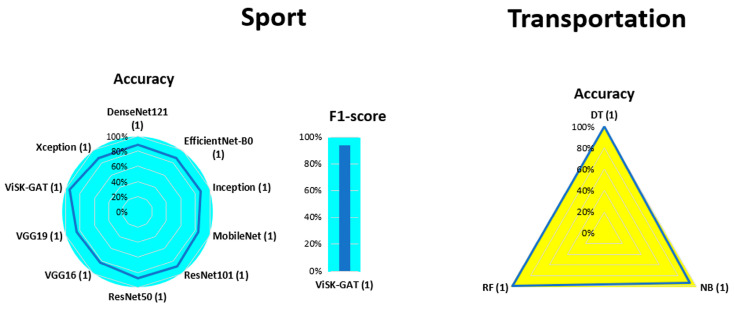
Performance analysis of different algorithms used in ML (yellow) and DL (blue) methods in Sport (**left**) and Transportation (**right**).

**Table 1:** Keyword combination for each database.

Database	Keyword Combinations
PubMed/Medline Google Scholar IEEE Xplore	posture AND (“artificial intelligence” OR AI) AND (“work-related musculoskeletal disorders” OR “WMSDs”) AND accuracy AND precision AND (“F1 score” OR F1-score) AND specificity AND sensitivity
ScienceDirect	posture AND AI AND WMSD AND accuracy AND precision AND F1-score AND specificity AND sensitivity

**Table 2:** Detailed presentation of context, methods, and algorithms for each of the 58 studies included.

Authors	Occupational Activity	Posture	Model	Objective	Method	Algorithms	Data Acquisition Method	Sensors Position	Number of Subjects Tested (Male/Female)
Kapse et al., 2024 [46]	Agriculture	General working posture	10 segments, 13 joints	Posture classification	ML, DL	MobileNet, ResNet, Inception, VGG-16, SVM, DT, RF, ANN	Camera	Ext	6
Abdel Hady et al., 2024 [86]	All	Standing	2 segments, 1 joint	Posture classification	ML, DL	OO, LSTM, CNN, RF, SVR, Bagging	Goniometer	Waist	60 (0/60)
Abdullah et al., 2025 [87]	All	Sitting	-	Posture classification	DL	CNN	RGB Camera	Ext	NA (70%/30%)
Dandumahanti et al., 2023 [88]	All	Sitting	-	Posture classification	ML	DT, KNN, SVM	EMG	Sternocleidomastoid and upper trapezius	30 (30/0)
Darvishi et al., 2017 [89]	All	General working posture	-	WMSD assessment from risk factors	ML	LR, KNN, NN	Questionnaire NASA-TLX	-	92
Hossain et al., 2023 [90]	All	General working posture	13 segments, 16 joints	Ergonomic posture risk assessment—REBA score	DL	DNN	Camera	Ext	NA
Hu et al., 2018 [91]	All	Standing	2 segments, 1 joint	Posture classification	DL	LSTM	Electromagnetic sensors, force plate	C7, T12, S1, Ext	44
Jiang et al., 2022 [92]	All	Sitting	-	Posture classification	ML	RF, LR, DT	Triboelectric nanogenerators	Chest	NA
Jiao et al., 2024 [93]	All	General working posture	13 segments, 16 joints	Ergonomic posture risk assessment—REBA score	DL	Encoder	Camera	Ext	9
Kim et al., 2018 [94]	All	Sitting	-	Posture classification	ML, DL	CNN, NB, MLR, DT, NN, SVM	Force sensing resistors	Seat of the chair	10
Kim et al., 2019 [95]	All	Sitting	-	Posture classification	DL	ANN, MNN, CNN	Force sensing resistors	Seat of the chair	26 (14/12)
Li et al., 2020 [96]	All	Manual handling	13 segments, 16 joints	Ergonomic posture risk assessment—RULA score	DL	CNN	Camera	Ext	12 (12/0)
Ogundokun et al., 2022 [97]	All	Human activity	-	Posture classification	DL	CNN, MLP	RGB camera	Ext	NA
Pereira et al., 2023 [98]	All	Sitting	-	Posture classification	ML	KNN, NC, SVM, GMM	ECG, Load cell	Seat of the chair, chest	22 (13/9)
Rodrigues et al., 2022 [99]	All	Sitting and standing	6 segments, 6 joints	Ergonomic posture risk assessment—RULA score	ML	RF	RBG and depth camera	Ext	20 (12/8)
Suárez Sánchez et al., 2016 [28]	All	General working posture	-	WMSD assessment from risk factors	ML	KNN	Questionnaire	-	11,054 (5917/5137)
Zhang et al., 2023 [100]	All	Sitting	-	Posture classification	DL	MMTM, GMU, DLF using RF, DLF using SVM, DLF using MLP, HOG + DT, HOG + KNN, HOG + RF	Temperature, pressure, infrared array sensors	Ext	20
Acharya et al., 2025 [47]	Construction	General working posture	-	Fatigue level assessment	ML, DL	LSTM, RF, XGBoost, CNN	EMG	Left and right erector spinae, rectus abdominis, rectus femoris, biceps femoris, tibialis anterior, gastrocnemius	NA
Antwi-Afari et al., 2018 [48]	Construction	General working posture	-	Posture classification	ML	ANN, DT, KNN, SVM	Foot plantar pressure	Foot	10 (10/0)
Antwi-Afari et al., 2020 [49]	Construction	Manual handling	-	WMSD assessment	ML	ANN, DT, KNN, RF, SVM	Foot plantar pressure, Accelerometer	Foot	2
Antwi-Afari et al., 2022 [32]	Construction	General working posture	-	Posture classification	DL	LSTM, Bi-LSTM, GRU	Foot plantar pressure, Gyroscope, Accelerometer	Foot	10 (10/0)
Seo et al., 2021 [50]	Construction	General working posture	-	Posture classification	ML	SVM	Kinect	Ext	8 (8/0)
Umer et al., 2020 [51]	Construction	Manual handling	-	Physical exertion assessment	ML	KNN, SVM, Discriminant, DT, Bagged Tree	ECG, Skin temperature, respiration	Thorax	10
Wang et al., 2021 [52]	Construction	Manual handling	13 segments, 13 joints	Ergonomic posture risk assessment—REBA score	DL	CNN	Camera	Ext	15 (9/6)
Xiahou et al., 2023 [53]	Construction	General working posture	21 segments, 14 joints	Posture classification	ML, DL	MLP, RNN, LSTM	Camera, pressure sensors, IMU, EEG	Ext, Foot, Head	7 (5/2)
Yang et al., 2020 [54]	Construction	Manual handling	-	Loading assessment	DL	Bi-LSTM	IMU, Camera	Ankle, Ext	12 (10/2)
Zhang et al., 2018 [55]	Construction	General working posture	10 segments, 7 joints	Posture classification	ML	BP-ANN, DT, SVM, KNN, EC	Camera	Ext	NA
Zhao et al., 2020 [56]	Construction	General working posture	5 segments, 4 joints	Posture classification	ML, DL	CLN, CNN, LSTM, SVM	IMU	Head, chest center, upper arm, thigh, and leg	4
Zhao et al., 2021 [57]	Construction	General working posture	5 segments, 4 joints	Posture classification	DL	CLN	IMU	Head, chest center, upper arm, thigh, and leg	9
Abdollahi et al., 2020 [58]	Healthcare	Standing	-	Posture classification	ML	SVM, MLP, K-mean	IMU, Wii balance	Sternum, Ext	94 (94/0)
Ferrone et al., 2021 [59]	Healthcare	General working posture	-	WMSD assessment from risk factors	ML	RF	Questionnaire	-	64 (14/50)
Han et al., 2024 [60]	Healthcare	Manual handling	10 segments, 7 joints	Ergonomic posture risk assessment—REBA score	DL	ST-GCN, CNN	RGB camera	Ext	NA
Hartley et al., 2024 [61]	Healthcare	Standing	2 segments, 1 joint	Posture classification	DL	CNN	Optoelectronic motion capture system	Ext	83 (36/47)
Luo et al., 2024 [62]	Healthcare	General working posture	-	WMSD assessment	ML	LR, SVM, Enet, RF, XGBoost, MLP	Questionnaire	-	617 (214/403)
Sen et al., 2024 [63]	Healthcare	General working posture	-	Posture classification	ML	ERG-AI	Accelerometers	Knee, thigh, waist, upper back, arm	114
Thiry et al., 2022 [64]	Healthcare	Standing	2 segments, 1 joint	Posture classification	ML	NB, KNN, SVM, DT, RF, AdaBoost	IMU	T12, S2, Thigh	40
Tomkins-Lane et al., 2022 [65]	Healthcare	Standing, walking	-	Posture classification	ML	RF	Accelerometers	Right hip	117 (65/112)
Villalobos et al., 2022 [66]	Industry	Standing	-	Ergonomic posture risk assessment—RULA score	ML	ET, SVM, RF, DT	IMU	Wrist	20
Abobakr et al., 2019 [67]	Manufacturing	General working posture	16 segments, 15 joints	Ergonomic posture risk assessment—RULA score	DL	ResNet	IMU, depth and RGB camera	Ext	6 (6/0)
Conforti et al., 2020 [68]	Manufacturing	Manual handling	7 segments, 6 joints	Posture classification	ML	SVM	IMU	Sternum, Pelvis, Thigh, Shank, Foot	26
Cruciata et al., 2025 [69]	Manufacturing	Manual handling	-	Ergonomic posture risk assessment—RULA score	DL	SPECTRE-ViT	IMU, RBG camera	Full body, Ext	NA
Davoudi Kakhki et al., 2025 [70]	Manufacturing	Manual handling	-	WMSD assessment	DL	CNN, MLP, LSTM	EMG	Left and right deltoid, elevator scapulae, biceps brachii, flexor carpi radialis	25 (15/10)
Donisi et al., 2021 [71]	Manufacturing	Manual handling	-	Ergonomic posture risk assessment	ML	DT, RF, GB, AdaBoost, KNN, NB, MLP, SVM, LR	IMU	Waist	7
Huang et al., 2024 [72]	Manufacturing	Manual handling	13 segments, 13 joints	Ergonomic posture risk assessment—REBA score	DL	CNN	Camera	Ext	26 (20/6)
Matos et al., 2024 [73]	Manufacturing	Sitting	4 segments, 3 joints	Ergonomic posture risk assessment—RULA score	ML	SVM, NB	Optoelectronic motion capture system	Ext	12
Mudiyanselage et al., 2021 [74]	Manufacturing	Manual handling	-	Ergonomic posture risk assessment	ML	DT, SVM, KNN, RF	EMG	Thoracic and lumbar extensors muscles	1
Nath et al., 2018 [75]	Manufacturing	General working posture	-	Ergonomic posture risk assessment—OSHA score	ML	SVM	Smartphones	Arm, Waist	2 (2/0)
Prisco et al., 2024 [76]	Manufacturing	Manual handling	-	Posture classification	ML	SVM, DT, GB, RF, LR, KNN, MLP, PNN	IMU	Chest	15 (9/6)
Senjaya et al., 2023 [77]	Manufacturing	Manual handling	20 segments, 16 joints	Ergonomic posture risk assessment—RULA score	DL	DNN, Bi-LSTM, CNN, HBU, HyNet	Camera, Leap Motion	Ext	12
Su et al., 2023 [29]	Manufacturing	Sitting	16 segments, 13 joints	Ergonomic posture risk assessment—REBA score	ML	DT	Camera	Ext	11 (8/3)
Markova et al., 2024 [78]	Office	Sitting	9 segments, 7 joints	Posture classification	ML, DL	RF, DLNN, GB	Photography	Ext	100 (64/36)
Piñero-Fuentes et al., 2021 [79]	Office	Sitting	13 segments, 5 joints	Posture classification	DL	CNN	Camera	Ext	12
Roh et al., 2018 [80]	Office	Sitting	-	Posture classification	ML	SVM	Load cell	Seat of the chair	9
Sasikumar et al., 2020 [81]	Office	Sitting	-	Ergonomic posture risk assessment—RULA score	ML	RF, NB, NN, KNN, DT, SVM	Cameras, Questionnaire NMQ	Ext	66
Zemp et al., 2016 [82]	Office	Sitting	-	Posture classification	ML	SVM, MNR, NN, RF	Force sensing resistors, IMU	Backrest, armrest of the chair	41 (16/25)
Zemp et al., 2016 [83]	Office	Sitting	-	Posture classification	ML	RF	Pressure sensors	Seat of the chair	20 (13/7)
Rahman et al., 2025 [84]	Sport	Human activity	Multiple segments and joints (depending on sport)	Ergonomic posture risk assessment—REBA score	DL	VGG16, VGG19, ResNet50, ResNet101, InceptionV3, Xception, EfficientNet-B0, MobileNetV2, DenseNet121, ViSK-GAT	Camera	Ext	NA (70%/30%)
Hanumegowda et al., 2022 [85]	Transportation	General working posture	-	WMSD assessment from risk factors	ML	DT, RF, NB	Questionnaire	-	370 (370/0)

**Assessment tool:** NMQ = Nordic Musculoskeletal Disorders Questionnaire; OSHA = Occupational Safety and Health Administration; REBA = Rapid Entire Body Assessment; RULA = Rapid Upper Limb Assessment. **Sensor type abbreviation:** ECG = Electrocardiography; EEG = Electroencephalography; EMG = Electromyography; IMU = Inertial Measurement Unit. **Sensor position abbreviation:** Ext = Exterior sensor. **Method abbreviation:** DL = Deep Learning; ML = Machine Learning. **Algorithms abbreviation:** AdaBoost = Adaptive Boosting; ANN = Artificial Neural Network; Bi-LSTM = Bidirectional Long Short-Term Memory; BP-ANN = Back-Propagation Artificial Neural Network; CLN = Convolutional Long Short-Term Memory Network; CNN = Convolutional neural networks; DLF = Decision-level fusion; DLNN = Deterministic Learning Neural Network; DNN = Deep Neural Networks; DT = Decision Tree; EC = Ensemble classifier; ET = Extremely randomized Trees; GB = Gradient Boosted Tree; GMM = Gaussian Mixture Model; GMU = Gated Multimodal Unit; GRU = Gated Recurrent Unit; HBU = Hybrid network of Bi-LSTM and Unidirectional LSTM; HOG = Histogram of Oriented Gradients; KNN = K-Nearest Neighbors; LR = Logistic Regression; LSTM = Long Short-Term Memory; MLP = MultiLayer Perceptron; MLR = Multinomial Logistic Regression; MMTM = MultiModal Transfer Module; MNN = Multi-layer Neural Networks; MNR = Multinomial Regression; NB = Naïve Bayes Classifier; NC = Nearest Centroid; NN = Neural Network; OO = Optimized Optuna; PNN = Probabilistic Neural Network; RF = Random Forest; RNN = Recurrent Neural Network; ST-GCN = Spatial Temporal Graph Convolutional Networks; SVM = Support Vector Machine; SVR = Support Vector Regression. **Other:** NA = Not available.

**Table 3:** Performance parameters present in each of the 58 included studies.

Authors	Occupational Activity	Accuracy	Specificity	Sensitivity	Precision	F1-Score
Kapse et al., 2024 [46]	Agriculture	X		X	X	X
Abdel Hady et al., 2024 [86]	All	X		X	X	X
Abdullah et al., 2025 [87]	All	X	X	X		
Dandumahanti et al., 2023 [88]	All	X	X	X	X	X
Darvishi et al., 2017 [89]	All	X				
Hossain et al., 2023 [90]	All	X		X	X	X
Hu et al., 2018 [91]	All	X		X	X	X
Jiang et al., 2022 [92]	All	X				
Jiao et al., 2024 [93]	All			X	X	X
Kim et al., 2018 [94]	All	X		X	X	
Kim et al., 2019 [95]	All	X		X	X	
Li et al., 2020 [96]	All			X	X	X
Ogundokun et al., 2022 [97]	All	X				
Pereira et al., 2023 [98]	All	X		X	X	X
Rodrigues et al., 2022 [99]	All	X		X	X	X
Suárez Sánchez et al., 2016 [28]	All	X	X	X		
Zhang et al., 2023 [100]	All	X				X
Acharya et al., 2025 [47]	Construction	X				X
Antwi-Afari et al., 2018 [48]	Construction	X				
Antwi-Afari et al., 2020 [49]	Construction	X				
Antwi-Afari et al., 2022 [32]	Construction	X	X	X	X	X
Seo et al., 2021 [50]	Construction	X				
Umer et al., 2020 [51]	Construction	X				
Wang et al., 2021 [52]	Construction			X	X	X
Xiahou et al., 2023 [53]	Construction	X				X
Yang et al., 2020 [54]	Construction	X	X	X	X	X
Zhang et al., 2018 [55]	Construction	X				
Zhao et al., 2020 [56]	Construction					X
Zhao et al., 2021 [57]	Construction					X
Abdollahi et al., 2020 [58]	Healthcare	X	X	X		X
Ferrone et al., 2021 [59]	Healthcare	X				
Han et al., 2024 [60]	Healthcare	X				
Hartley et al., 2024 [61]	Healthcare	X	X	X		X
Luo et al., 2024 [62]	Healthcare	X	X	X		
Sen et al., 2024 [63]	Healthcare	X		X	X	X
Thiry et al., 2022 [64]	Healthcare	X				
Tomkins-Lane et al., 2022 [65]	Healthcare	X	X	X		
Villalobos et al., 2022 [66]	Industry	X		X	X	X
Abobakr et al., 2019 [67]	Manufacturing	X				
Conforti et al., 2020 [68]	Manufacturing	X	X	X	X	
Cruciata et al., 2025 [69]	Manufacturing	X		X	X	X
Davoudi Kakhki et al., 2025 [70]	Manufacturing	X		X	X	X
Donisi et al., 2021 [71]	Manufacturing	X	X	X		
Huang et al., 2024 [72]	Manufacturing			X	X	X
Matos et al., 2024 [73]	Manufacturing					X
Mudiyanselage et al., 2021 [74]	Manufacturing	X				
Nath et al., 2018 [75]	Manufacturing	X		X	X	X
Prisco et al., 2024 [76]	Manufacturing	X	X	X	X	X
Senjaya et al., 2023 [77]	Manufacturing					X
Su et al., 2023 [29]	Manufacturing	X				
Markova et al., 2024 [78]	Office	X		X	X	X
Piñero-Fuentes et al., 2021 [79]	Office	X				
Roh et al., 2018 [80]	Office	X				
Sasikumar et al., 2020 [81]	Office	X		X	X	X
Zemp et al., 2016 [82]	Office	X				
Zemp et al., 2016 [83]	Office	X			X	
Rahman et al., 2025 [84]	Sport	X				X
Hanumegowda et al., 2022 [85]	Transportation	X				

## Data Availability

The original contributions presented in this study are included in the article. Further inquiries can be directed to the corresponding author.

## Supplementary Materials

The following supporting information can be downloaded at: https://www.mdpi.com/article/10.3390/bioengineering13030298/s1, PRISMA 2020 for abstract checklist.

## Abbreviations

The following abbreviations are used in this manuscript:

AdaBoost	Adaptive Boosting
ANN	Artificial Neural Network
Bi-LSTM	Bidirectional Long Short-Term Memory
BP-ANN	Back-Propagation Artificial Neural Network
CLN	Convolutional Long Short-Term Memory Network
CNN	Convolutional neural networks
DL	Deep Learning
DLF	Decision-level fusion
DLNN	Deterministic Learning Neural Network
DNN	Deep Neural Networks
DT	Decision Tree
EC	Ensemble classifier
ECG	Electrocardiography
EEG	Electroencephalography
EMG	Electromyography
ET	Extremely randomized Trees
Ext	Exterior sensor
GB	Gradient Boosted Tree
GMM	Gaussian Mixture Model
GMU	Gated Multimodal Unit
GRU	Gated Recurrent Unit
HBU	Hybrid network of Bi-LSTM and Unidirectional LSTM
HOG	Histogram of Oriented Gradients
IMU	Inertial Measurement Unit
KNN	K-Nearest Neighbors
LR	Logistic Regression
LSTM	Long Short-Term Memory
ML	Machine Learning
MLP	MultiLayer Perceptron
MLR	Multinomial Logistic Regression
MMTM	MultiModal Transfer Module
MNN	Multi-layer Neural Networks
MNR	Multinomial Regression
MSD	Musculoskeletal Disorders
NB	Naïve Bayes Classifier
NC	Nearest Centroid
NMQ	Nordic Musculoskeletal Disorders Questionnaire
NN	Neural Network
OO	Optimized Optuna
OSHA	Occupational Safety and Health Administration
PNN	Probabilistic Neural Network
REBA	Rapid Entire Body Assessment
RF	Random Forest
RNN	Recurrent Neural Network
RULA	Rapid Upper Limb Assessment
ST-GCN	Spatial Temporal Graph Convolutional Networks
SVM	Support Vector Machine
SVR	Support Vector Regression
WMSD	Work-Related Musculoskeletal Disorders
